# Dealing with Emotional Vulnerability and Anxiety in Nurses from High-Risk Units—A Multicenter Study

**DOI:** 10.3390/ijerph19095569

**Published:** 2022-05-04

**Authors:** Esther Arimon-Pagès, Paz Fernández-Ortega, Núria Fabrellas-Padrés, Ana María Castro-García, Jaume Canela-Soler

**Affiliations:** 1 Hospital Clinic Barcelona, Faculty of Health Sciences, University of Barcelona (UB), 08036 Barcelona, Spain; esterarimon@gmail.com (E.A.-P.); nfabrellas@ub.edu (N.F.-P.); 2 Faculty of Health Sciences, Campus Bellvitge, University of Barcelona (UB), 08036 Barcelona, Spain; 3 Institut Català d’Oncologia Barcelona, Grupo de Investigación Enfermera GRIN, Instituto de Investigación de Biomédica de Bellvitge (IDIBELL), 08908 Barcelona, Spain; 4 Primary Care Nurse, Servicio Madrileño de Salud, 28020 Madrid, Spain; anamariacastrogarcia@gmail.com; 5 Department of Basic Clinical Practice, Faculty of Medical and Health Sciences, University of Barcelona (UB), 08036 Barcelona, Spain; jcanela@ub.edu; 6 Department of Epidemiology and Biostatistics, College of Public Health, University of South Florida, Tampa, FL 33612, USA

**Keywords:** compassion, anxiety, compassion fatigue, burnout, secondary traumatic stress, nursing care

## Abstract

Compassion fatigue and anxiety derived from continued exposure to trauma and death greatly impact nurses’ quality of care and quality of life, increasing their desire to leave work. The aim of the study is to assess compassion fatigue and anxiety prevalence and their association with secondary variables. A multicenter, cross-sectional study in nurses from four high-risk units, Emergency, Intensive Care, Oncology, and Pediatrics, was carried out in 14 hospitals in Catalonia (Spain) between 2015 and 2016. The primary endpoints were compassion satisfaction and compassion fatigue (burnout and secondary traumatic stress), which were assessed by Professional Quality of Life (ProQOL), and anxiety, assessed with the State-Trait Anxiety Inventory (STAI). Multivariable logistic regression analyzed the association of sociodemographic, training, working, and psychological factors. Of a total of 1302 nurses, 18.6% presented low compassion satisfaction; 19.7%, high burnout; and 36.4%, high secondary traumatic stress. Trait anxiety scored high in 7.2%. Although compassion satisfaction was present, it did not protect sufficiently against the high level of compassion fatigue or anxiety present in nurses in all centers. The working conditions in the units and variables showed a strong association with nurses’ desire to leave. This corroborates the global challenge of healthcare professionals’ shortage. Participants expressed the need for better training in emotional management.

## 1. Introduction

Compassion was defined by Kanov [1] as “a relational process that involves noticing the other person’s pain, experiencing an emotional reaction and acting in some way to help or alleviate the pain”. Compassion constitutes one of the cornerstones of deontology in the nursing profession and an essential characteristic of quality nursing care [2,3]. The act of caring brings great satisfaction to nurses, but the constant exposure to suffering, illness, and death of patients can cause negative emotions and make them vulnerable to disorders such as anxiety [4] and compassion fatigue (CF) [5]. 

The construct “compassion fatigue” was coined by Joinson [6] in 1992 to describe a particular form of burnout in nurses, differentiating between the pressure that any work environment can exert, such as excessive loads, overexertion, or lack of recognition, which defines burnout, and the emotional affectation derived from contact with trauma, which defines CF. The first theoretical model defined by Figley [7,8], based on this concept, described adaptive or non-adaptive responses to stress, defining it as “the cost of caring”, and used the term CF interchangeably with secondary traumatic stress (STS). According to this conceptual framework, later, Stamm [9] grouped STS and burnout in compassion fatigue (CF) and defined the Professional Quality of Life Scale (ProQOL) as the balance between negative aspects of care, CF, and positive aspects of care, compassion satisfaction (CS).

Compassion fatigue is a disorder with signs and symptoms similar to those of post-traumatic stress. “Empathic distress” or aversive response and anxiety [4] are final results defined as an unpleasant feeling of discomfort, worry, apprehension, or fear [10], which are all feelings of threat. In these cases, they are at higher risk of physical and psychological disorders or adopting unhealthy lifestyles with self-medication, substance abuse, alcohol, and tobacco. At work, there are strong links to medication errors, a desire to leave the profession, and work absenteeism [11].

When nurses are affected by these negative aspects, the demands from working conditions exceed their own skills and resources, and they may react by immersing themselves in stress and anxiety, creating a protective barrier between patients and themselves. The result is the suppression of nurses’ altruistic behavior, an emotional withdrawal of patient with a reduction in empathic commitment and added negative feelings [12]. The development of these disorders compromises not only their well-being, but also the quality of their care [4,12]. 

The hospital environment itself acts as a stressor [13] that can interact with other external sources of stressors, such as the rising demands on professionals’ time, efficiency, and productivity [14]. This production line approach to care [15] can also undermine the compassion and the proximity necessary to perform optimal caring acts, creating conflicts in professional values and causing stress, anxiety, and frustration. Despite these conceptual differences between STS and burnout, both syndromes include emotional disturbances and tend to appear together [5,10]. 

There is a lot of controversy around the co-existence of positive (CS) and negative aspects (STS and burnout) in nurses due to the inverted relationship between them [16], and both aspects, positive and negative, compensate for each other. Both conditions affect nurses’ health and are good predictors of quality of care and patient satisfaction [17,18]. 

Some hospital settings may increase the vulnerability of nurses due to their distinctiveness: patients experiencing life-threatening diseases and high vulnerability, great complexity, elevated technification, shortage of time in a critical care unit [19,20], unexpected death, trauma, and violence experienced by patients in emergency departments [21,22]. In oncology units, a high physical and emotional demand, repeated exposure to pain and death, and moral dilemmas are distinctive [23]. Painful procedures, serious injuries, or death in children are risk factors in pediatrics [24]. In these environments, a nurse’s focus oscillates between the patient’s well-being, the functioning of machines added to the manager’s staff, and families’ expectations, devaluating their reflection and critical thinking [25].

Accordingly, nurses’ prevalence of secondary traumatic stress and burnout can vary in global estimations depending on the unit. Thus, CF and STS have been focused on primarily in nurses working in special units around the world due to their emotional suffering. Studies on emergency nurses in China [21], on Jordanian critical care nurses [26], and a systematic review of oncology nurses [23] and pediatric nurses [27] show a high prevalence of CF in nurses, and these results are not very different in Europe [20]. Anxiety has also been studied [4,28,29] and has been found to be associated with suppressing altruistic behavior, distancing from the patient, and reducing their empathic commitment [29].

For healthcare organizations, CF in nurses triggers absenteeism, turnover, and negative performance in their duties [30]. These issues have broader implications for healthcare staff retention, certainly one of the major global challenges today [31]. The high prevalence of CF and burnout, combined with low CS, directly impact emotional involvement in work and quality of life, affecting the quality of care and raising healthcare costs. It is critical to identify both individual and working factors to develop training and preventive strategies, especially in hospital units with high exposure to risk factors.

## 2. Methods

### 2.1. Hypothesis and Aims

We hypothesized that compassion fatigue and anxiety are high in nurses in emergency, intensive care, oncology, and pediatric units and are related to personal, training, and psychological factors.

The aims of this study were: (1) to assess the nurses’ emotional vulnerability, expressed with CS, CF (burnout and STS) prevalence, and anxiety in nurses working in high-risk units, and (2) to describe associations between these prevalent outcomes and demographic, educational, work-related, and psychological factors in nurses working in intensive care, emergencies, oncology, and pediatric units.

### 2.2. Design

An observational, descriptive, cross-sectional, multicenter study was carried out using three self-report questionnaires with nurses working in highly emotional environments at hospitals from January 2015 to February 2016.

### 2.3. Setting and Participants

The participants were nurses from 14 hospitals belonging to the National Health System of Catalonia, Spain, selected by convenience and by similarity criteria. Of these, 10 were university hospitals, and the rest were regional hospitals. Inclusion criteria for participants were to be a nurse belonging to any of the following departments: Emergency Department, Intensive Care Unit (ICU), Oncology Unit, or Pediatrics Unit. We excluded nurses on leave or in training at the time of the study.

The sample size calculation was based on the maximum indetermination assumption, with a confidence level of 95% and a precision of ±5% for subgrouping by type of unit. Based on these calculations, each subgroup needed 289 participants. The relative strength of the sample was established by calculating a total number of participants of 1156, but 1302 nurses were finally recruited. The mean rate of participation by team was 80%. Altogether, 1500 questionnaires were distributed. The mean rate of participation by team was 80%.

### 2.4. Data Collection

For ad hoc content validation of the questionnaire, we undertook a pilot survey with 47 nurses in the Emergency Department to gauge understanding and the time needed for completion; no changes were necessary in the questionnaire. One nurse per center was assigned and trained as a referent; these professionals hosted orientation meetings for nurses working in each unit, shift, and center to explain the study objectives and recruit participants. Each participant received on paper a copy of the validated questionnaires (Professional Quality of Life (ProQOL) [9] and the State-Trait Anxiety Inventory (STAI) [32]), plus a purpose-designed questionnaire, an information sheet, and an informed consent form, which were individually answered. Data were recorded by means of voluntary self-report, and we sent several reminders and collected the completed forms from participants at various time points, as long as it was necessary to achieve a minimum participation of 80%.

### 2.5. Variables and Instruments

The main outcomes were to assess nurses’ vulnerability through dependent variables such as compassion satisfaction, secondary traumatic stress, and burnout measured with the ProQOL scale, and anxiety with the STAI Inventory Scale.

#### 2.5.1. Professional Quality of Life Scale (ProQOL)

The Spanish version of the ProQOL scale has three independent dimensions and 30 items related to professional quality of life [9]. Positive quality of life aspects were determined by the 10 items of the compassion satisfaction domain, the CS subscale (Cronbach’s α = 0.87). Negative aspects were determined by 10 items of the burnout subscale, expressed in means (Cronbach’s α = 0.72), plus the 10 items of the secondary traumatic stress subscale (Cronbach’s α = 0.80). According to the author’s instructions, the two last subscales measure CF altogether.

Respondents could choose 5-point Likert options for each item, from “never” to “always”. Cut-off points are established by the author for every subscale, determining three risk levels for each dimension: low, moderate, and high.

#### 2.5.2. State-Trait Anxiety Inventory (STAI)

The STAI scale [32] comprises 20 items for each of two subscales: trait anxiety, defined as individual differences that predispose people to anxiety, and state anxiety, defined as subjective feelings of tension, apprehension, nervousness, and worry at a given time point. For the Spanish version, Cronbach’s α is 0.85 and 0.92 for the respective subscale.

We categorized scores from both subscales into the three risk levels established by the author, as we did with ProQOL (low, moderate, and high), and we also re-expressed the STAI score on a continuous scale from 0 to 100.

#### 2.5.3. Participant’s Characteristics

Additional variables were collected through a purpose-designed questionnaire and included demographic details (age, sex, number of dependents), training (in the specialized areas, emotional management training), work-related variables (hospital unit, work shift: morning shift, afternoon shift, and first or second night shift or rotating, weekly workload, years of experience, years in current position), and psychological indicators (perceived need for emotional management training, desire to change units, and feelings of regret/satisfaction regarding chosen profession).

### 2.6. Data Analysis

Qualitative variables are presented as percentages and quantitative variables as means (± standard deviation (SD)). We used prevalence and 95% confidence intervals (CIs) as a frequency measure. We analyzed associations between variables through binary logistic regression, choosing variables based on univariable analysis and previous studies in the literature. We expressed estimates as adjusted odds ratios (ORa) and 95% CIs. We considered p values of less than 0.05 to be statistically significant. All statistical analyses were performed using SPSS v.20 (IBM Inc., Chicago, IL, USA) for Windows.

### 2.7. Rigor and Validity

The ProQOL and STAI questionnaires have been validated in the Spanish language and are widely used and supported in the literature. We piloted the questionnaires used to detect and correct any errors in the study design and its instruments. The recruited participants in the different units exceeded the minimum required size according to the power calculation for every unit (*N* = 289), and the total sample (*n* = 1302) was diverse and representative of hospitals across Catalonia. The 14 participating hospitals were distributed across the whole geographical area.

### 2.8. Ethical Considerations

The Clinical Research Ethics Committees of all participating centers approved the study. Each participant received written and oral information on the study objectives and an invitation to participate on a voluntary and anonymous basis, in compliance with Spanish law 15/1999 of 13 December on data protection, which aims to ensure appropriate confidentiality and data protection.

## 3. Results

### 3.1. Participants’ Characteristics

We received completed questionnaires from 1302 nurses. Participant characteristics are summarized in Table 1: the respondents were primarily women, and half had dependent family members. Nurses working the night shift were the most frequent responders, and on average, they had completed a third of their working life, with over half of their career spent in their current unit. One-fifth had received specialized training in their work unit. Two-thirds had never received any training in emotional management, although practically all respondents perceived a need for it. Half of them admitted that they had considered transferring to another unit, a quarter that they had contemplated changing professions, and a fifth that they would not choose nursing again if they had the choice.

### 3.2. Compassion Satisfaction, Burnout, Compassion Fatigue and Anxiety

Overall, about one in five professionals reported a low level of CS; one in six, a high level of burnout; and one in three, a high level of STS. Trait anxiety was high in 7.2% of the respondents and state anxiety in 11.8% (Table 2). These were measured quantitatively; the mean state anxiety was 9.0 points higher than trait anxiety (36.9 versus 27.9 on a scale of 0 to 100).

### 3.3. Analysis of the Outcome Variable: Multivariable Logistic Regression

Table 3 shows the factors associated with different subscales according to multivariable logistic regression. Low CS was associated with having considered a change in professions and regret about choosing nursing. Those who worked rotating shifts were more satisfied. A high level of burnout was associated with having considered transferring to another unit, changing professions, and regretting their career choice. On the other hand, high burnout was less common in nurses who worked the afternoon and the rotating shifts, those who worked fewer than 20 h per week, and those who had received emotional management training. A high level of STS was associated with being a woman, having considered transferring to another unit, changing professions, and regretting their career choice.

A high level of state anxiety was associated with having considered transferring to another unit, changing professions, and regretting their career choice (Table 4). With regard to trait anxiety, high levels were associated with working in the emergency department, having considered transferring to another unit, changing professions, and regretting their career choice.

## 4. Discussion

The high prevalence of CF in our study confirms our initial hypothesis regarding the CF prevalence in nurses according to Mooney [33]. Our results are comparable to or a little lower than those of Yu et al.’s results in emergency nurses [21], Al Barmawi’s [26] in critical care nurses, oncology nurses in a systematic review [23], or Roney’s [27] in pediatric nurses, and in this case, the positive factors associated with work did not compensate the negative ones.

More than 60% of our participants scored moderate/high state anxiety levels. The 9% difference between trait and state anxiety may be directly attributable to the anxiety generated by the work environment. Sydenham et al. [4] questioned how the negative aspects of care are related to emotional avoidance and, therefore, to withdrawal from the patient.

Few factors were associated with the ProQOL and STAI subscales. Sociodemographic, training, and work-related characteristics do not appear to be major predictive or protective factors compared to the studies by Alharbi [34], who related CF to gender or age, and Jakimowicz [35], who related CF to training. Rather, the work unit seems to be the most important to determining nurses’ vulnerability in our study, which is consistent with the results reported by Andriani [24] and Mooney [33].

Both components related to CF, burnout, and STS were highly prevalent in our sample, separately. This reveals the importance of exploring how to cope with the factors related to each one and their interactions. Some studies have highlighted the direct role of organizational and environmental factors [36], such as resource availability, teamwork, professional recognition, and positive feedback on burnout, but these variables also appear to be related to STS, and this may indicate the possibility that burnout would be a precursor of STS [37]. This link, which could explain the joint appearance of both conditions in our results, is important because the desire to quit was strongly associated with low scores in the domain of compassion satisfaction and high scores in anxiety, burnout, and especially STS [19]. Half the participants expressed an urge to leave their service, which coincides with Roth [38], and a quarter wanted to quit the profession altogether, compared to the 56% reported by Lee [39]. Regardless of differences related to training, culture, dependent family members, and environment, the desire to quit appears repeatedly as a constant dimension of international importance [38,40], and this is perhaps the most relevant finding of our study.

Adequate emotional management is positively related to empathy and quality of care, whereas lower resilience has been associated with maladaptive behavior, emotional aversion, and increased anxiety [4]. A third of our participants reported receiving emotional management training, a variable significantly associated with lower burnout in both our study and in the study by Shoji [37], who reported that specialized training for caregiving in emotionally complex situations and for self-efficacy reduced burnout and STS.

The absence of previously reported data in our country, the clustering of high-risk units, and the high number of participating centers and professionals have allowed us to generate the knowledge needed to establish a preliminary vision of the problem and to lay a foundation for future studies in the area. The prevalence obtained illustrates a clear need for greater institutional awareness on this essential issue, its impact, and its consequences for both professionals and citizens. These have implications for nurses’ quality of life, the quality of care that patients and populations receive, and institutional costs. Future studies identifying factors that mediate the development of CF (burnout and STS) will increase understanding of the disorder, facilitating the implementation of appropriate training and preventive and supportive activities. Although there are no published examples of interventions for CF in our setting, evidence in other countries has described effective measures that provide resilience-promoting resources and skills [41]. The institutional strategies designed to increase personal resilience in nurses and decrease levels of self-reported psychological symptoms could be an initial approach to tackle this important problem.

The current pandemic situation related to COVID-19 has made us wonder how this factor will impact the mental and emotional health of nurses. Despite the overexposure to death in some areas, such as emergencies and critical care, it does not appear that the affectation of these nurses is greater than in the previous situation, and nurses continue to prioritize the well-being of the patient over their own [34]. A study in health professionals that stay close to patients with COVID-19 in Spain showed that in all professionals, there is a medium/high level of burnout and ETS, which is a comparatively stable degree compared to the previous situation, and comparable to results in the present study [42]. On the other hand, anxiety shows how a construct on it has increased not only interest, but also the prevalence in nurses [43,44]. Focused on understanding and addressing the sources of anxiety in health care professionals, a paper published in JAMA, USA during COVID-19 summarized their concerns in five requirements: “hear me, protect me, prepare me, support me and care for me” [45].

## 5. Limitations and Future Directions

Cross-sectional studies do not meet the temporality criterion to establish cause and effect. Moreover, we cannot rule out the possible existence of personal, professional, or institutional confounders that we did not control for in the analysis. There may have also been some self-selection bias, with an overrepresentation of professionals who were sensitive to the phenomenon of CF compared to those who were not. The number of participants and centers is high and representative of the study setting, but the external validity is limited to hospitals with similar profiles and environments.

Nurses’ lack of awareness and their difficulties in caring for suffering patients and their families can severely affect them with high CF and anxiety and compromise the health system. This panorama implies the need for personal traits reinforcement [46], educational strategies [47], and institutional policies [19].

## 6. Conclusions

STS and burnout constitute CF and show similar prevalence results. The associations between CF and personal, training, and work-related variables did not permit us to firmly establish a professional profile that is especially vulnerable to CF and anxiety, although their prevalence in all units is high. This high prevalence in nurses in all high-risk units in our results points to the work environment as the main risk factor.

Compassion satisfaction and fatigue—that is, positive and negative aspects of nursing care—tend to appear together, as do the syndromes of burnout (an environmental factor) and STS (a factor related to exposure to trauma), although the sociodemographic, training, and work environment factors studied did not seem to be determinants. Emotional management training was associated with lower levels of burnout and was perceived as necessary by virtually all participants. STS was closely related to nurses’ desire to leave their unit and profession in all the units studied.

The development of personal coping tools and institutional prevention and support policies should be established as a priority, especially for professionals at the highest risk.

## Figures and Tables

**Table 1:** Description of professional participants.

Variable	% (*n*/*N*) or Mean ± SD
Age (years)	37.7 ± 10.3
Women	87.1% (1133/1301)
Dependents	49.6% (637/1285)
Working unit	
Emergency department	24.1% (314/1302)
ICU	30.4% (396/1302)
Oncology	22.7% (296/1302)
Pediatrics	22.7% (296/1302)
Work shift	
Morning	20.8% (270/1300)
Afternoon	20.2% (263/1300)
Night	35.8% (466/1300)
Rotating	23.2% (301/1300)
Specialized training	20.8% (269/1291)
Professional work experience (years)	14.3 ± 9.9
Experience in current unit (years)	8.1 ± 10.0
Weekly workload	
<20 h	8.6% (111/1286)
20–40 h	80.0% (1029/1286)
>40 h	11.4% (146/1286)
Prior training in emotional management	35.8% (463/1295)
Perceived need for emotional management training	97.1% (1260/1298)
Has considered changing units	49.8% (639/1283)
Has considered changing professions	25.5% (318/1247)
Would choose the nursing profession again	79.3% (1007/1270)

ICU: intensive care unit; SD: standard deviation.

**Table 2:** Prevalence and severity of professional quality of life indicators.

Scale and Subscale	*n*	Prevalence (%)	95% CI
*Professional Quality of Life questionnaire*			
Compassion Satisfaction			
*Low*	242	18.6	16.5–20.7
*Moderate*	685	52.6	48.9–55.3
*High*	375	28.8	26.3–31.3
Burnout			
*Low*	211	16.2	14.2–18.2
*Moderate*	835	64.1	61.5–66.7
*High*	256	19.7	17.5–21.7
Secondary traumatic stress			
*Low*	156	12.0	10.2–13.8
*Moderate*	672	51.6	48.9–54.3
*High*	474	36.4	33.8–39.0
*State-Trait Anxiety Inventory*			
Trait anxiety			
*Low*	683	52.9	50.2–55.6
*Moderate*	515	39.9	37.2–42.6
*High*	93	7.2	5.8–8.6
State anxiety			
*Low*	544	42.0	39.3–44.7
*Moderate*	597	46.1	43.4–48.8
*High*	153	11.8	10.1–13.6

CI: confidence interval.

**Table 3:** Factors associated with CS, burnout, and STS, according to the Professional Quality of Life (ProQOL) questionnaire.

Characteristics	Low Compassion Satisfaction		High Burnout		High STS	
	Prevalence % (*n/N*)	ORa (95% CI)	Prevalence % (*n/N*)	ORa (95% CI)	Prevalence % (*n/N*)	ORa (95% CI)
Sex						
Women					37.4% (424/1133)	1
Men					29.8% (50/168)	0.68 (0.47–0.8)
Work shift						
Morning	22.6% (61/270)	1	24.1% (65/270)	1		
Afternoon	16.3% (43/263)	0.67 (0.41–1.10)	17.9% (47/263)	0.58 (0.37–0.83)		
Night	23.8% (111/466)	1.08 (0.72–1.62)	21.9% (102/466)	0.80 (0.54–1.19)		
Rotating	8.6% (26/301)	0.36 (0.21–0.62)	13.3% (10/301)	0.50 (0.31–0.81)		
Weekly workload						
<20 h			9.0% (10/111)	1		
20–40 h			21.1% (217/1029)	0.81 (0.52–1.23)		
>40 h			17.8% (26/146)	0.51 (0.29–0.89)		
Emotional management training						
No			21.8% (181/832)	1		
Yes			16.6% (74/463)	0.66 (0.47–0.92)		
Has considered transferring to another unit						
No			12.0% (77/644)	1	29.0% (187/644)	1
Yes			27.5% (175/639)	1.99 (1.43–2.77)	44.0% (281/639)	1.46 (1.12–1.90)
Has considered changing professions						
No	12.8% (119/929)	1	15.1% (140/929)	1	31.0% (288/929)	1
Yes	36.8% (117/318)	2.22 (1.56–3.18)	34.0% (108/318)	1.88 (1.31–2.69)	51.9% (165/318)	1.75 (1.28–2.40)
Would choose the nursing profession again						
Yes	11.0% (111/1007)	1	15.5% (156/1007)	1	32.1% (323/1007)	1
No	45.2% (119/236)	0.22 (0.15–0.31)	33.8% (89/263)	0.59 (0.41–0.85)	51.3% (135/263)	0.58 (0.42–0.80)

CI: confidence interval; ORa: adjusted odds ratio; STS: secondary trauma stress. Statistical significance set at *p* < 0.05.

**Table 4:** Factors associated with anxiety, according to State-Trait Anxiety Inventory (STAI).

Characteristics	High State Anxiety		High Trait Anxiety	
	Prevalence % (*n/N*)	ORa (95% CI)	Prevalence % (*n/N*)	ORa (95% CI)
Working unit				
Emergency department			11.9% (37/311)	1
Intensive care			6.1% (24/392)	0.43 (0.24–0.77)
Oncology			5.4% (16/297)	0.50 (0.26–0.96)
Pediatrics			5.5% (93/1291)	0.50 (0.26–0.97)
Has considered transferring to another unit				
No	6.7% (43/640)	1	4.2% (27/640)	1
Yes	16.4% (104/636)	1.56 (1.02–2.38)	10.3% (65/633)	1.78 (1.04–3.05)
Has considered changing professions				
No	7.5% (69/924)	1	4.1% (41/921)	1
Yes	24.4% (77/315)	2.20 (1.42–3.42)	14.2% (45/316)	2.10 (1.23–3.58)
Would choose the nursing profession again				
Yes	7.5% (75/1001)	1	5.1% (51/998)	1
No	26.8% (70/261)	0.36 (0.23–0.55)	15.3% (40/261)	0.45 (0.27–0.77)

CI: confidence interval; ORa: adjusted odds ratio. Statistical significance set at *p* < 0.05.

## Data Availability

The data that support the findings of this study are available from the corresponding author upon reasonable request.
